# Linac performance benchmarking: Feasibility of a web‐based tool for comparison of local machine performance check (MPC) results with the global community

**DOI:** 10.1002/acm2.70892

**Published:** 2026-10-28

**Authors:** Lauren May, Michael P. Barnes, Michael Pearson, Benjamin Zwan, José Antonio Baeza‐Ortega, Benjamin Cooper, Mohammad Hussein, Ajeet Mishra, Shiaw Juen (Eugene) Tan, Jackson Zifodya, Peter B. Greer, Catharine H. Clark, Joerg Lehmann

**Affiliations:** ^1^ Department of Radiation Oncology Calvary Mater Hospital Newcastle New South Wales Australia; ^2^ School of Science University of Newcastle Newcastle New South Wales Australia; ^3^ Centre for Medical and Radiation Physics University of Wollongong Wollongong New South Wales Australia; ^4^ Medical Physics Department Guys and St Thomas’ Hospital London UK; ^5^ School of Biomedical Engineering & Imaging Sciences King's College London London UK; ^6^ Central Coast Cancer Centre Gosford Hospital Gosford New South Wales Australia; ^7^ Medical Physics and Radiation Engineering Canberra Hospital Garran Australian Capital Territory Australia; ^8^ Department of Radiation Oncology King Khalid University Medical City Abha Saudi Arabia; ^9^ Central West Cancer Care Centre Orange Health Service Orange New South Wales Australia; ^10^ North West Cancer Centre Tamworth Rural Referral Hospital Tamworth New South Wales Australia; ^11^ Radiotherapy and Radiation Dosimetry Group National Physical Laboratory Teddington UK; ^12^ Department of Radiotherapy Physics University College London Hospital London UK; ^13^ Department of Medical Physics and Bioengineering University College London London UK; ^14^ National Radiotherapy Trials Quality Assurance Group University College London Hospital London UK; ^15^ Institute of Medical Physics University of Sydney Sydney New South Wales Australia

**Keywords:** inter‐center comparison, MPC, quality assurance

## Abstract

**Background:**

Varian machine performance check (MPC) is a manufacturer integrated quality control system included as standard for TrueBeam and Halcyon linear accelerators (linacs) to assess performance using a broad range of geometric and beam tests. MPC is a highly standardized method of performance testing, is generally free of inter‐user variability and is quick and easy to perform allowing daily acquisitions. Therefore, previous studies have highlighted the potential of comparative analysis of MPC data in investigating differences in performance of individual linacs and identifying outliers. Statistical process control (SPC) is an established approach commonly used to assess stability and behavior of sequential data and identify standard operating ranges which has previously been applied to radiotherapy quality assurance data and MPC data.

**Purpose:**

This study aimed to investigate the feasibility of a web‐based platform using SPC and aggregated MPC data to allow users to compare their machine performance against that of a cohort of others in the radiotherapy community.

**Methods:**

A previously developed web‐based platform for radiotherapy data sharing was expanded to include the MPC data collection and comparison tool. Pilot centers exported data from their MPC applications and uploaded it through center‐specific portals. Within the application, SPC analysis was used to calculate upper control limits (UCL), lower control limits (LCL) and central lines (CL) for the data sets of each individual linac as well as cohort averages. Interactive plots of longitudinal data for individual machines were displayed for users and overlaid with SPC limits for the specific machine and the cohort averages. Additional analyses were conducted externally from the app where the average and range of SPC control limits for the cohort were compared against MPC tolerances.

**Results:**

MPC data for 57 648 measurements taken between April 2016 and May 2026 were collected across seven centers from 21 TrueBeam linacs (15 with Millennium multi‐leaf collimator (MLC), six with HD120 MLCs) and one Halcyon. Variation in performance for specific tests was identified for individual machines compared against the cohort as well as for individual MLC leaves for specific machines. Across the cohort of TrueBeam linacs, average control ranges for all geometric tests were within MPC thresholds. However, the range of values for UCL and LCL exceeded tolerances for relative gantry angle. Machines installed before 2021 were found to have slightly larger variability in mean MLC offset with an average control range width of 0.20 mm and 0.18 mm for bank A and B, respectively, compared to machines installed after 2021 of 0.10 mm and 0.13 mm. Analysis of data for individual linacs segmented per year showed a change in mean MLC offset occurring around 2021.

**Conclusions:**

The usefulness and feasibility of a tool to compare MPC data against the radiotherapy community was demonstrated. Inter‐center comparison of data allowed trends to be identified which would otherwise not be observable for individual centers. Further development and distribution of the MPC data sharing and comparison tool may provide improved confidence and understanding of machine performance and assist low‐resourced clinics in delivering safe and accurate radiotherapy treatments.

## 1 INTRODUCTION

Varian (Siemens Healthineers AG, Forchheim, Germany) machine performance check (MPC) is a fully‐integrated tool for linear accelerator (linac) performance assessment available on all Varian TrueBeam linacs from version 2.0 and Varian Halcyon linacs, for routine performance testing and acceptance testing.^1^ The MPC system acquires a series of automated kilovoltage (kV) and megavoltage (MV) images both with and without the vendor supplied Isocal phantom in place across varying states of machine operation to assess both beam and mechanical component performance.^1^Its standard daily test suite includes geometry checks evaluating the accuracy of the mechanical axes of the linac including: isocenter size and MV and kV imager offsets, positioning accuracy of the collimation system including individual MLC leaves and MLC banks, jaws and rotational offsets, gantry and couch positioning/rotational accuracy. Beam checks include output constancy, beam uniformity and beam center shift.^1^ Most centers using MPC carry out the tests daily. It is mandated on the Halcyon platform that all tests must have passed before treatment can commence.^2^ The performance of MPC has been evaluated in multiple studies^1^, ^3^, ^4^, ^5^, ^6^, ^7^, ^8^, ^9^, ^10^, ^11^, ^12^, ^13^ which in general have been positive as to MPC's performance as a daily check device, while also highlighting limitations.

Statistical process control (SPC) is an established approach for quality control used to determine stability and behavior of sequential data.^14^, ^15^, ^16^ SPC calculates standard operating ranges where variability is attributed to uncontrollable uncertainties, within which data values are considered to be “in‐control”.^15^, ^16^ SPC control charts are often used for visualization where plotted sequential data is overlaid with a center line (CL) representing the standard data point value along with an upper control limit (UCL) and lower control limit (LCL) derived statistically. This type of analysis can be useful for radiotherapy quality assurance (QA) data, which typically includes a degree of random variation due to measurement uncertainties and innate fluctuations in processes. Several radiotherapy specific studies have utilized SPC analysis to determine when systematic changes occur, and QA results are no longer ‘in‐control' allowing intervention or machine maintenance to be performed.^17^, ^18^, ^19^, ^20^, ^21^, ^22^ Identifying ‘out‐of‐control' processes early can allow intervention to happen prior to machine QA tolerances being exceeded, preventing unscheduled downtime and potentially interrupted patient treatments, whilst also potentially allowing the machine to be maintained to tighter tolerances without additional maintenance.^20^, ^21^

Several studies have applied SPC to MPC data^21^, ^23^ including one study which compared MPC data across multiple sites.^24^ However, the potential to do this on a large scale has not been fully explored. Analyses have also been extended to include machine‐state monitoring and demonstrated the potential for predictive QA using MPC data to determine when processes will exceed clinical tolerances.^21^, ^25^, ^26^ As suggested by Puyati et al.^21^ and recommended by an IPEM topical report (2025),^27^ the standardized and user independent nature of manufacturer integrated quality control (MIQC) data, of which MPC is an example, would allow the direct statistical comparison of individual treatment units against other matching unit types internationally. Having access to such information could be expected to provide individual users enhanced confidence in their machine performance and also help identify outlier machines for potential investigation. Such analysis could be expected to be particularly beneficial in helping assure safe and accurate radiotherapy for isolated and low resourced centers. However, there is no current infrastructure solution available which would allow this kind of intercenter analysis, including flexibility for the addition of new users, and customizable up‐to‐date benchmarking analyses.

This study's specific aim was to investigate the feasibility of a web‐based platform using SPC and aggregated MPC data to allow users to compare their machine performance against a cohort of others in the radiotherapy community. Additionally, this study aimed to report findings from analyses of the initial cohort of machines. The global aim was to provide improved confidence and understanding of machine performance through data sharing amongst the radiotherapy community and to assist low‐resourced clinics in delivering safe and accurate radiotherapy treatments.

## 2 METHODS

### 2.1 Web platform development

A previously developed in‐house Python Flask web application deployed in Microsoft Azure was used as the foundation of the tool. It was initially designed to support distribution and upload of digital imaging and communications in medicine (DICOM) files and data collection via completion of forms for a previous international study.^28^ The web application includes secure individualized portals for each center including two‐factor authentication via Azure email services. The user base already included 128 centers from 26 countries. For this study, the application was extended with coding assistance from Claude (Anthropic PBC) to include the MPC data collection and comparison tool. Claude was used to generate first drafts of hypertext markup language (html) templates which were manually edited. Python code segments required to perform SPC calculations and data presentation were also generated by Claude and manually edited where necessary and integrated into the existing application code. All code segments were carefully read and understood by an experienced coder, and randomly selected outputs including the number of included data points and SPC statistics were visually inspected and confirmed to align with expected results. This included a user interface to allow the MPC data upload and comparison as well as an administrative interface for data curation. Access to the tool was granted to a subset of selected pilot centers from existing research collaborations.

### 2.2 MPC data collection

An instruction document was provided to participating centers and is included as supplementary materials. MPC daily measurement data was exported by participating centers using the standard export function in the MPC application, which generates cumulative historical data in the form of comma separated values (csv) files. Depending on software configuration, some systems export cumulative data since install dates while some export approximately two years of measurement history along with baseline measurements.

Participants accessed the web application through their departments’ portal and uploaded their csv files together with details for the machine model, beam energy, machine serial number and machine ID. Upon upload, the tool performs preliminary checks on the data upload including identifying if the beam energy from the file name matches the selected energy and if data has previously been uploaded for the machine. As MPC data is cumulative, additional uploads to an existing machine include some duplicate data followed by the new measurements. During the automated preliminary checks, duplicate data is identified by matching the machine serial number, energy and test date and time to existing database entries. A summary of upload details and any relevant warnings are provided to the user to preview before confirming the upload. All uploaded csv files are retained in the web application file storage, while only new, non‐duplicative MPC data are added to the database.

### 2.3 Statistical process control analysis and data visualization

The tool calculates control chart values for the entire measurement history of each machine individually, with the UCL, LCL and CL calculated as per equations 1, 2 and 3, respectively:

(1) $$UCL\;\;=\;\;\;\bar{x}+E\times \bar{MR}$$

(2) $$LCL\;=\;\;\bar{x}\;-\;E\times \bar{MR}$$

(3) $$CL\;=\;\;\bar{x}\;$$

where $\bar{x}$ is the average of *m* data observations, $\bar{MR}$ is the average of the moving range as in equation 4, and E is shown in equation 5:

(4) $$\bar{MR}\;=\;\frac{\Sigma \left|x_{i}\;-\;x_{i-1}\right|}{m\;-\;1}$$

(5) $$E\;\;=\;\;\;\frac{3}{d_{2}}=2.66$$

where d_2_ is Hartley's constant for a moving range calculated over two adjacent data points.^16^ This form of SPC analysis has previously been successfully used by several studies for MPC data.^21^, ^23^, ^24^

In the tool, control charts are visualized for the user with individual data points plotted and overlaid with the UCL, LCL and CL for the selected machine. In addition, UCL, LCL and CL values are averaged across all machines in the database with matching machine and MLC model to the selected machine and overlaid onto the plot to allow visual comparison of the individual machine against the cohort average. All data comparisons against the cohort are fully anonymized and only averaged cohort data is shown, not data for individual centers or machines. Numerical values are displayed for the control range (difference between the UCL and LCL) for the individual machine as well as the average control range for the cohort. For tests which include data for individual MLC leaves, a separate plot is generated which shows the UCL, LCL, and CL of each MLC leaf for both banks for the selected machine only.

The average UCL and LCL, along with limits (maximum UCL and minimum LCL), were calculated across all TrueBeam linacs for: isocenter size, MV and kV imager offsets, positioning accuracy of the jaws, rotational offsets and couch positioning accuracy. These were also calculated for beam center shift, beam output constancy, and beam uniformity for 6MV, 6MV flattening filter free (FFF), 10MV and 10MV FFF beam energies. For MLC mean offset, data were separated by MLC model before calculating the same metrics. Averages and limits were compared against the predefined MPC thresholds for each test. Mean MLC offset was calculated for each of the TrueBeam linacs with Millennium MLC using only data from 2024 onwards to reduce differences in data collection periods. For a subset of TrueBeam linacs with Millennium MLC, mean MLC offset data were separated by year and SPC limits calculated for each annual period.

### 2.4 Feasibility analysis

Feasibility was assessed based on the following criteria: (1) users are able to successfully upload data for TrueBeam and Halcyon linacs, (2) these data are stored in the database and able to be accessed for analysis, and (3) users are able to view comparison results for their machines. Open‐ended user feedback was collected via email from participants. Suggestions for improvements and additional features were collated.

## 3 RESULTS

### 3.1 MPC data collection

For this feasibility study, MPC data was collected across seven pilot centers from 21 TrueBeam linacs (15 with Millennium MLC, six with HD120 MLCs). Data from one Halcyon machine was collected to confirm the web interface upload functionality. However, further analyses were restricted to the TrueBeam linacs. Corresponding beams from the same centers were beam matched. The earliest measurement data were collected in April 2016 and most recent data was from May 2026. Data for 9 TrueBeam linacs (8 with Millennium MLC, 1 HD120 MLC) from a single center were cumulative from install date, while remaining machine data included approximately the two most recent years of measurement data (with the exception of one linac installed in 2025) (Table S1, supplementary materials). In total, data was uploaded for 57 648 measurements which equated to ∼5.5 million individual data points.

Users were successfully able to view plots of their data compared against SPC statistics calculated from the cohort. User feedback is summarized in Table S2 in supplementary materials.

### 3.2 Statistical process control analysis and data visualization

Variations in machine performance for specific tests were identified across the cohort (Figure 1). This included the presence of outlying values in otherwise consistent data for some machines and tests, such as the example shown in Figure 1a of the gantry relative parameter. Trends over time were also identified such as repeated drift and correction of beam output as shown in Figure 1b and distinct step changes in MLC mean offset for bank A as shown in Figure 1c. Variation in performance across MLC leaves were also identified through the web application interface as exemplified in Figure 2 which shows that, for the presented linac, some of the wider MLC leaves (leaves 1–10 for bank A and leaves 51–56 for bank B) had a control range of approximately double that of the other leaves for each bank respectively. Outlying leaves identified through this visualization in the application interface are able to be selected individually for comparison against the cohort values.

**FIGURE 1:**
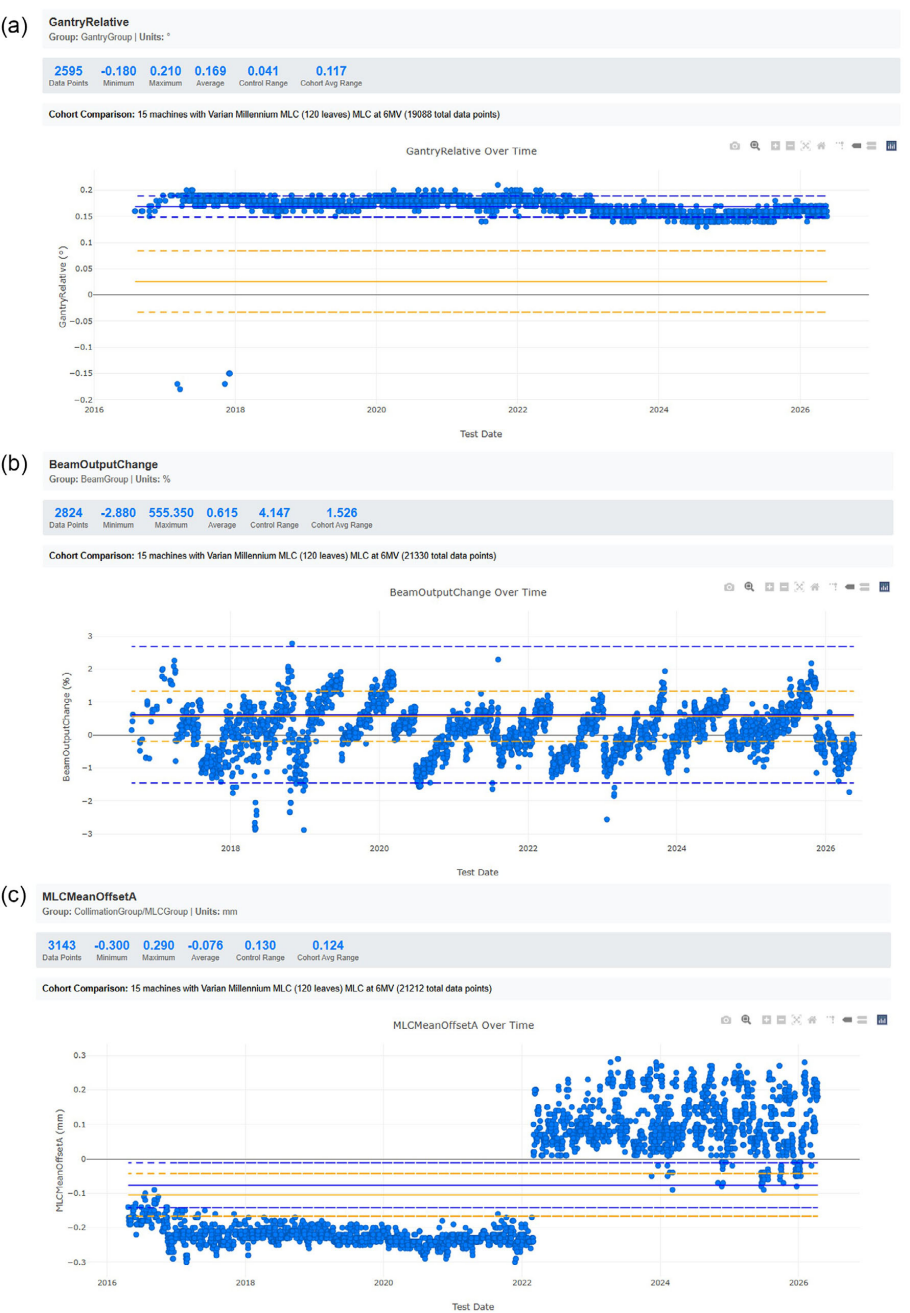
Three separate screen captures showing example machine performance data from one center, as displayed in the web application, showing a) gantry angle relative offset, b) beam output change and c) MLC mean offset for bank A. Individual MPC data points for the user's selected machine are shown in blue, the center line (solid blue) and the upper and lower control limits (dashed blue lines) for this machine are also shown. The average center line value for matching linac models in the cohort are shown in solid orange lines and the average upper and lower control limits in dashed orange lines.

**FIGURE 2:**
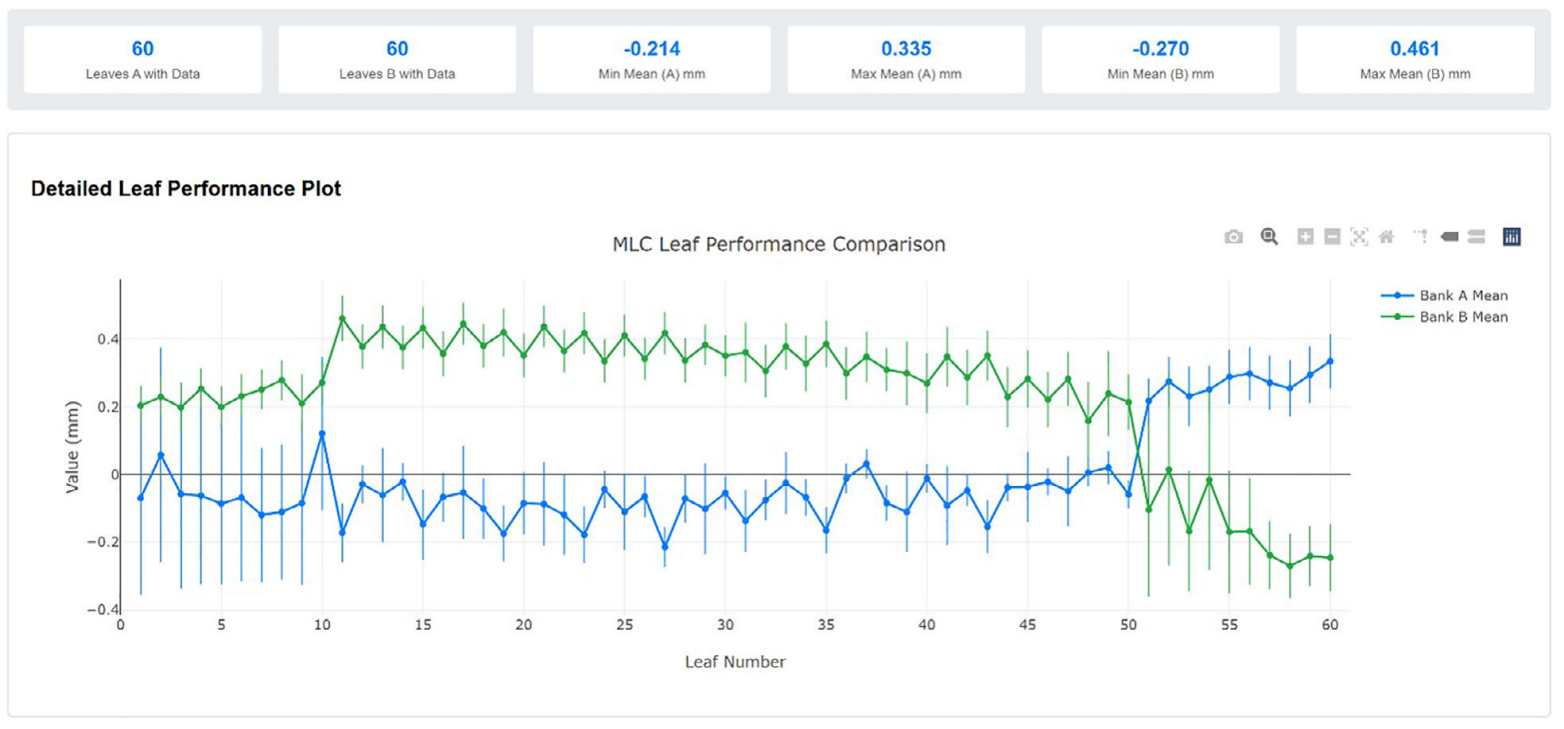
Example individual Millennium MLC leaf data as displayed in the web application, showing mean values and upper and lower control limits (vertical bars) for each MLC leaf (banks A and B) for the selected machine.

For all presented geometric MPC tests, the average control range (difference between average UCL and average LCL) for the cohort of TrueBeam linacs was within the MPC thresholds (Figure 3). For all geometric tests except for the gantry angle relative position (maximum UCL = 0.34, minimum LCL = −0.31), the maximum UCL and minimum LCL across all linacs were also within the MPC thresholds. The consistency of the MLC position was similar for the Millennium MLC and HD120 MLC, again with the average control ranges and maximum values across all linacs within the threshold values (Figure 3). For beam center shift, beam output change and beam uniformity (Figure 4), although the average upper and lower control limits were all within tolerances, there is variation across machines with some individual machines exceeding the threshold for beam output change and beam constancy. The maximum UCL for beam output change was 2.67%, 7.68% and 2.30% for 6MV, 6FFF and 10MV beams respectively. The minimum LCL was −4.04% and −2.44% for 6FFF and 10MV beams and the maximum UCL for 10FFF beam uniformity was 2.60%.

**FIGURE 3:**
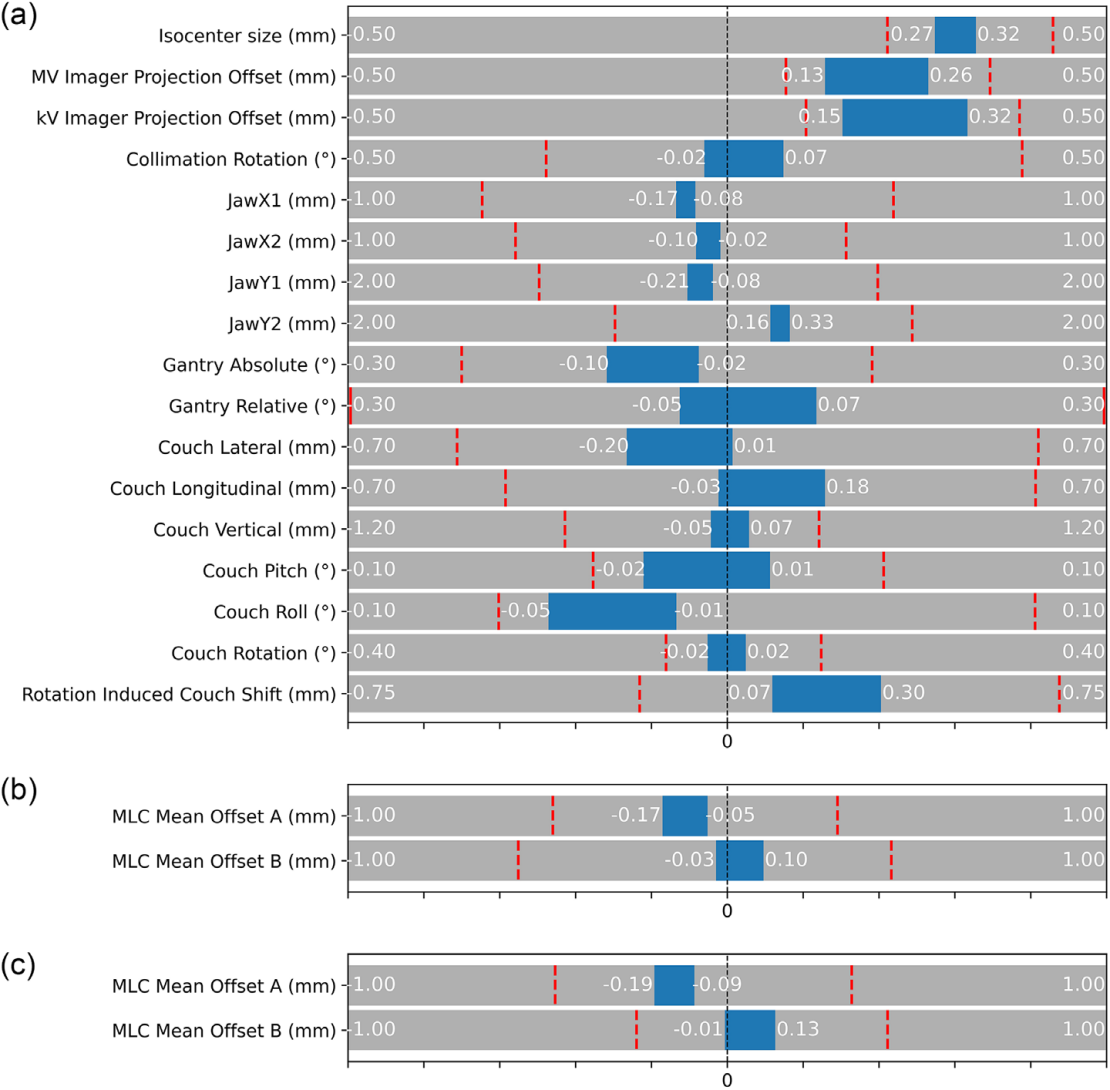
The range between average upper and average lower control limits (in blue) across the cohort of (a) all TrueBeam linacs, (b) TrueBeam linacs with millennium MLCs and (c) TrueBeam linacs with HD120 MLCs against the predefined MPC threshold values for each test (right and left axis limits). The maximum UCL and minimum LCL are shown in red dashed lines, with a red solid line at the axis limit for the gantry relative parameter indicating the maximum UCL value (0.34) and minimum LCL (−0.31) exceeded the threshold and are not shown.

**FIGURE 4:**
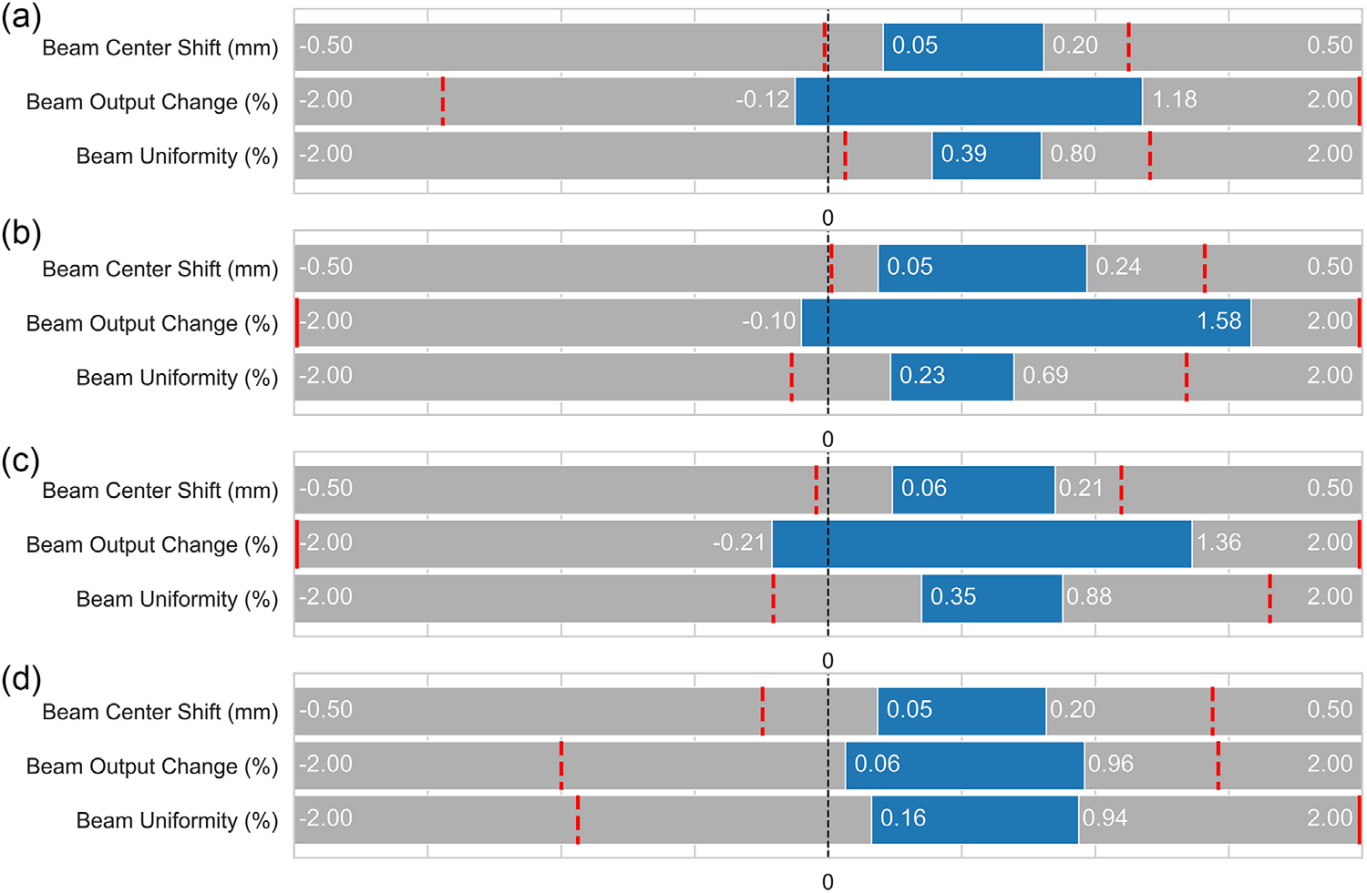
The range between average upper and average lower control limits (in blue) for beam tests across the cohort of TrueBeam linacs for (a) 6MV (*n* = 21), (b) 6FFF (*n* = 16), (c) 10MV (*n* = 16) and (d) 10FFF (*n* = 15). The maximum UCL and minimum LCL are shown in red dashed lines, with a red solid line at the axis limit indicating the value exceeded the threshold and is not shown.

The variation across machines is further shown in Figure 5 for mean MLC offset for both banks for Millennium MLCs. There was variation in both the width of the control ranges as well as overall central line offset. Some machines displayed a narrow control range but with an overall offset approaching or even exceeding the current clinical recommended tolerance of 0.50 mm. Older machines had slightly wider control ranges where machines installed in 2020 or earlier had an average control range width for bank A of 0.20 mm which was twice that of machines installed after 2021 of 0.10 mm. This was less pronounced for bank B where the average control range width for older machines (pre‐2021) was 1.80 mm compared to 1.3 mm for newer machines (post‐2021). As shown in Figure 6, a shift can be observed in the central line position for the three oldest linacs occurring in approximately 2021, after which the control range width increases.

**FIGURE 5:**
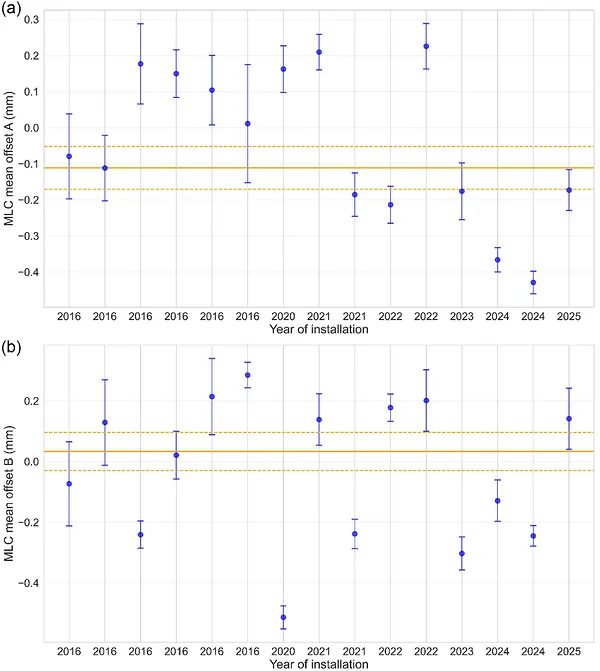
Comparison of MLC mean offset data collected from 2024 onwards for (a) bank A and (b) bank B between each TrueBeam linac with Millennium MLC. The central line value is indicated by a blue marker and upper and lower control limits represented by the error bars. The cohort average central line (solid horizontal line) and the average cohort upper and lower control limits (dashed lines) are also shown.

**FIGURE 6:**
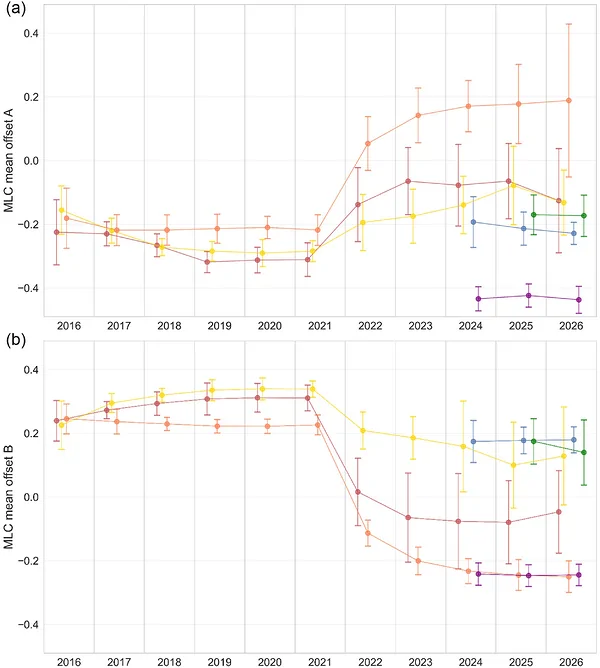
MPC data for the 3 earliest (orange, yellow and red) linacs, each installed in 2016, and 3 most recent linacs installed in 2024 (blue and purple) and 2025 (green) for MLC mean offset for (a) bank A and (b) bank B. SPC limits are shown for each individual linac per year with the central line indicated by the marker position and upper and lower control limits represented by the error bars.

## 4 DISCUSSION

This study presents a proof of concept for a data analysis and comparison tool utilizing MPC data. Although a previous study has compared MPC data across several centers,^24^ this is the first study that demonstrates a scalable platform developed to allow users to compare their MPC data to relevant data of peers. Initial testing with data for 22 linacs across seven centers demonstrated how large‐scale, inter‐center analysis can enable identification of variations in machine performance between centers and potential trends over time.

While available commercial software includes some similar features such as time analysis of longitudinal data, SPC analysis and automatic upload of MPC data, the current tool demonstrates feasibility for comparison of data against the cohort of other linacs whenever the user chooses. As discussed in both a 2025 IPEM topical report^27^ and another study Puyati et al.,^21^ the standardization and user independence of MIQC data such as MPC allows direct statistical comparison of linacs of matching models internationally. Collaboration amongst the radiotherapy community has been identified as one pathway to overcome some barriers to offering complex radiotherapy treatments, particularly at rural and isolated centers.^29^ This comparative analysis can provide valuable information about the performance of individual linacs and thereby confidence in machine performance, which is an important step toward safely implementing complex delivery techniques. For example, users might perform MPC daily throughout their commissioning process, then before transitioning to clinical use, upload and assess the relative performance of their machine against the cohort of other users.

For the cohort of linacs presented here, the average control range for all geometric tests and beam checks were within the MPC thresholds (Figures 3 and 4). However, for some tests (specifically gantry relative, beam output change and beam uniformity) the maximum UCL or minimum LCL exceeded the threshold indicating the calculated standard operating range for at least one machine exceeded the threshold. In some cases, this intermachine variability may be due to non‐suitability of the SPC statistical methods applied here for certain data types as discussed below. However, such a result may also be real and a true indication of an outlier machine requiring investigation and hence demonstrating the utility of the tool. It should also be mentioned that the included pilot centers were chosen from existing collaborations which may represent relatively well‐resourced centers. Hence, these trends may not be representative of a larger cohort of centers. A notable difference in control ranges for mean MLC offset was also identified whereby older linacs had a slightly larger control range than newer linacs (Figure 5). In this case, the effect was more apparent for the MLC A side than the B side. Differing variability in mean MLC offset between bank A and bank B have similarly been observed by Barnes et al, 2025^4^. In Barnes's study the effect was not observed when checked against an independent method suggesting that the effect is specific to the MPC measurement rather than to the linac. It can be observed in Figure 6 that there was a sudden change in mean MLC offset for the presented linacs at around 2021, after which the width of the control range for these linacs was larger than it was prior to this change. This is also evident in the MLC mean offset data for bank A for an individual machine shown in Figure 1c. This timing coincides with when Varian changed linac install procedure at around 2020–2021 to introduce a 0.4 mm MLC gap when MLC leaves are nominally closed. The purpose of this procedural change was to minimize MLC collisions resulting in worn T‐nuts and hence MLC backlash which has been shown to result in MLC positional error.^30^ As such, the change could be expected to result in less variable MLC positioning. Accompanying the procedure change was a change in MPC whereby this gap could be entered as the MPC gap offset, with some dependence on software version. However, this would be expected to change the average rather than the spread of data. Further investigation into whether these introduced changes relate to the results presented in Figure 5 and Figure 6 is necessary as well as consideration of how potential procedural changes may impact SPC analyses and comparisons.

Some processes such as output drift present challenges for SPC analysis due to the repeated pattern of drifting in one direction then being adjusted, as shown in Figure 1b. This is evidenced in Figure 4 where the maximum UCL and minimum LCL calculated using SPC analysis for the beam output exceeded the thresholds for 6MV, 6FFF and 10MV energies, which is unlikely to be a true representation of clinically used machines, especially for the 6FFF beam where the maximum UCL for beam output change was 7.68%. In addition to difficulties in analyzing beam data using SPC, the occurrence of outlying values and maintenance events for other parameters needs to be considered. Strategies to minimize the effect of outlying results can be simple such as only including data within a set number of standard deviations. However caution must be taken when doing this so as to not discard genuinely outlying data which may be indicative of an underlying issue. Data curation may be required to exclude such data from cohort averages. Though, with future collection of data to increase the cohort size, the impact of outlying or atypical values will be reduced. As shown in Figure 1a, there are several individual data points which are clearly outliers despite the majority of data being very consistent. Additionally, in Figure 1c, a dramatic shift in results occurs after approximately 2022. It can be assumed that there was likely a maintenance event at this time and as such SPC analysis calculated using all data would overestimate the variation in performance. Since maintenance and re‐calibration are regular activities in radiation therapy, corresponding data patterns are expected. Hence, more complex and context specific analysis solutions beyond standard SPC are being investigated for future implementation. This may include the exponentially weighted moving average (EWMA) chart where more recent data is weighted higher over older data. This method has previously been applied to radiotherapy data and may be more sensitive to slow drifts of a process and achieve stable control limit calculations with fewer data points.^18^, ^19^, ^22^ Cumulative sum (CUSUM) charts have also been applied to radiotherapy data providing information about deviation from target values and being very sensitive to small changes.^22^ Also, correlation of QA results to service events to provide clear and specific detectability of maintenance related changes would complement this initiative.

A current area of interest for MPC data analysis is predictive QA. When a process becomes out‐of‐control, or exceeds either the UCL or LCL, there is generally a period before it will exceed the clinical tolerance.^21^ This is illustrated in Figure 3, where for each of the presented tests the average control range across the cohort of included linacs is within the MPC threshold. This can act as a ‘warning’ period where machine maintenance can be scheduled and performed to limit down‐time and interference with patient treatments. Predictive QA has the potential to allow time estimates of how long this warning period will last and therefore inform the urgency of machine maintenance,^21^, ^31^ but also to extend the warning period by predicting when a process will become out‐of‐control.^32^

Various analytical methods of predictive QA have been applied to machine QA data including MPC and Sun Nuclear DailyQA3 measurements: autoregressive integrated moving average (ARIMA),^21^, ^32^ Kalman Filter and Extended Kalman Filter models.^25^, ^33^ As with SPC analyses, the repeated drifts and corrections typical of beam output change data presents issues for predictive QA using simpler analyses such as ARIMA modelling. Some of these issues have been addressed in the analytical methods presented in Reynard et al.^25^ which makes use of an Extended Kalman Model. This kind of predictive QA could potentially be incorporated into future implementations of the tool to further improve its use, however there are some limitations that need to be addressed. One is that predictive QA is most beneficial with daily measurement data. Currently data upload to the platform is manual which would need to be improved upon.

While significant effort will be spent on further developing data analysis techniques, other additions to the tool will include visual indication of data in relation to either MPC thresholds or clinical tolerances such as recommended in consensus guidelines.^34^ It may also be useful for users to be able to configure tolerances shown to match their departmental practices. Conversely, users may wish to use data provided by the application to set local warning level tolerances. MPC data includes a large volume of individual tests, therefore, a visual indicator of when data is approaching within 10% of the threshold or exceeding the proposed limits is planned to be added. In addition, improved data curation abilities for users will be included such as editing and deleting previous submissions. In the current implementation, attempts were made to ensure the validity of data such as providing previews for csv uploads detailing the number of new data submissions and any pre‐existing data. The csv file name was also checked for the correct beam energy which is included as standard when exported from the Varian MPC app. However, data validation checks for the machine serial number are still being developed as this is not included in the csv files and relies on user entry. Expansion is also planned to include filtering options so that linacs can be compared against subsets of the cohort including within the same region or regulatory jurisdiction and against multiple linacs from one's own center which may be useful in assuring on‐going beam matching.

MPC has a number of strengths making it ideal for inter‐center comparison using SPC, including: MPC is generally free of user variability, it is a highly standardized method of QA and is quick and easy to perform making it suitable for daily data acquisitions.^21^ There may also be the opportunity to expand to other MIQC systems, such as Elekta's AQUA, or linac‐independent quality assurance systems. The presented comparison of MPC data from 21 TrueBeam linacs is currently the largest multicenter comparison reported in the literature. There is potential for this tool to expand far beyond that to include many more centers internationally. The tool was released to a limited number of selected pilot centers from the overall application's existing user base. However, with further development it is planned to be released to all users.

## 5 CONCLUSION

Feasibility was demonstrated for a tool to compare linac MPC data against the radiotherapy community. The usefulness of such a tool has previously been expressed in the literature but was demonstrated for the first time here through assessing inter‐linac variability and identifying average and outlying test values from a cohort of 22 linacs across seven centers. The comparison of linacs across centers allowed for identification of variation between linacs and potential trends over time which likely would not be possible for local comparisons of fewer machines. Future advancements and improvements for the tool will include improved analysis of certain data types such as beam output drift, implementation of predictive QA as well as allowing users to compare their linacs against the international radiotherapy community, local community and within their own center. Further development and distribution of the MPC data sharing and comparison tool may provide improved confidence and understanding of machine performance and assist low‐resourced clinics in delivering safe and accurate radiotherapy treatments.

## AUTHOR CONTRIBUTIONS

Lauren May: Developed the web application software, conducted data analysis and presentation, and primarily wrote the original draft manuscript. Michael P. Barnes: Proposed the idea for the project, provided scientific input into the data analysis, contributed machine data, and contributed significantly to writing and editing of the original draft manuscript. Michael Pearson: Contributed machine data, provided scientific input into the data analysis and provided review of the draft manuscript. Benjamin Zwan: Was involved in planning of the project, contributed machine data and provided review of the draft manuscript. José Antonio Baeza‐Ortega: Was involved in planning of the project and provided review of the draft manuscript. Benjamin Cooper: Contributed machine data and provided review of the draft manuscript. Mohammad Hussein: Provided review of the draft manuscript. Ajeet Mishra, Shiaw Juen (Eugene) Tan and Jackson Zifodya: Contributed machine data and provided review of the draft manuscript. Peter B. Greer: Was involved in planning of the project and provided review of the draft manuscript. Catharine H. Clark: Provided review the draft manuscript. Joerg Lehmann: Obtained funding for the project, managed the project and contributed significantly to editing and review of the manuscript.

## CONFLICT OF INTEREST STATEMENT

Joerg Lehmann is a consultant and auditor for the Australian Clinical Dosimetry Service (ACDS) and the consulting QA physicist for the Trans‐Tasman Radiation Oncology Group (TROG).

## ETHICS STATEMENT

No human participants, identifiable private information, or biospecimens are included in this study.

## Supporting information




Supporting Information

Supporting Information

## Data Availability

The data that support the findings of this study are available from the corresponding author upon reasonable request.
