# Supplementary material for: Linac performance benchmarking: Feasibility of a web‐based tool for comparison of local machine performance check (MPC) results with the global community
Source: J Appl Clin Med Phys. 2026 Oct 28;27(11):e70892. doi: 10.1002/acm2.70892 (PMC13715505; doi:10.1002/acm2.70892)
Supplement: Supplementary file 1 [file ACM2-27-e70892-s002.pdf]

Uploading MPC Data

From your centre's home page you will see an MPC data comparison module:

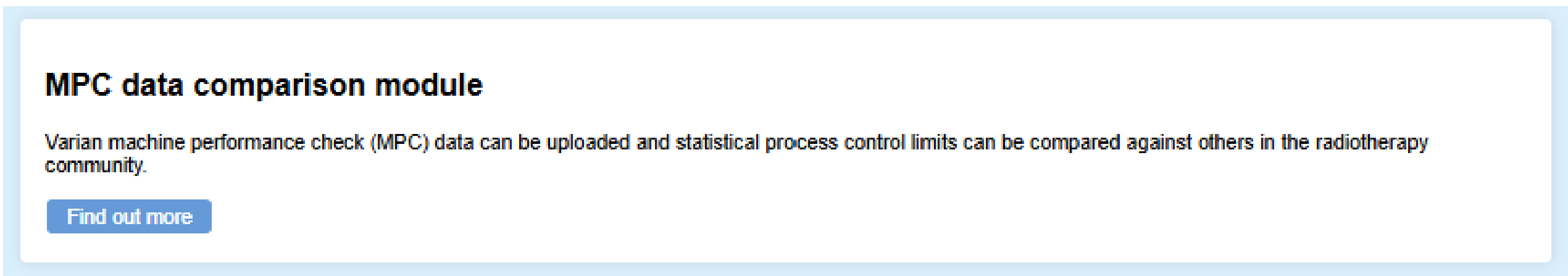

Click **find out more** to go to the MPC home page which will look something like this:

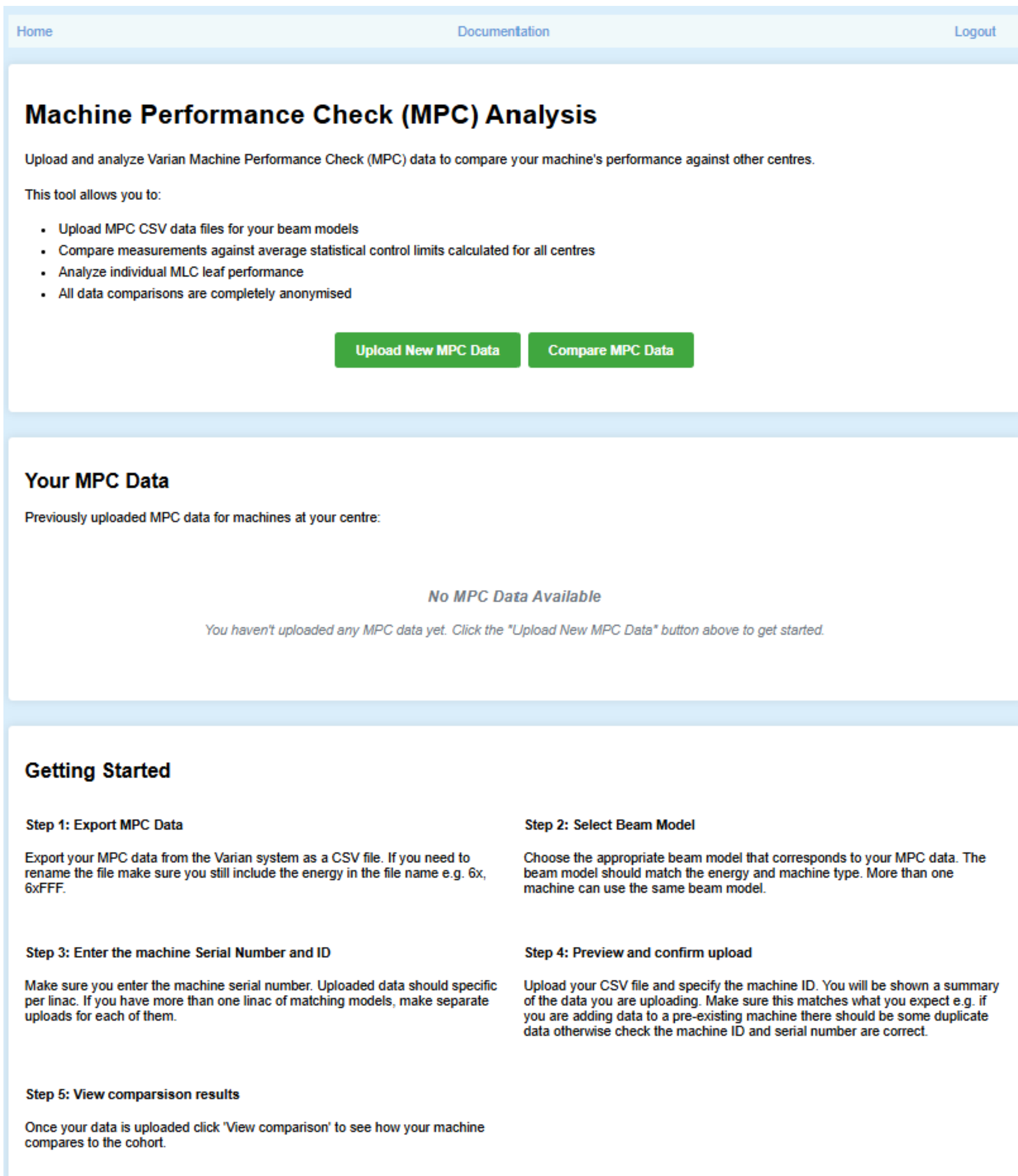

You will need to export your MPC data from your Varian system as csv files. If you need to rename the files, make sure to keep the energy in the file name e.g. '6x' or '6xFFF' etc. Also make sure to take note of the machine serial number as you will need to enter this when you upload data.

For your first upload click the **Upload New MPC Data** button. The page will then look like this:

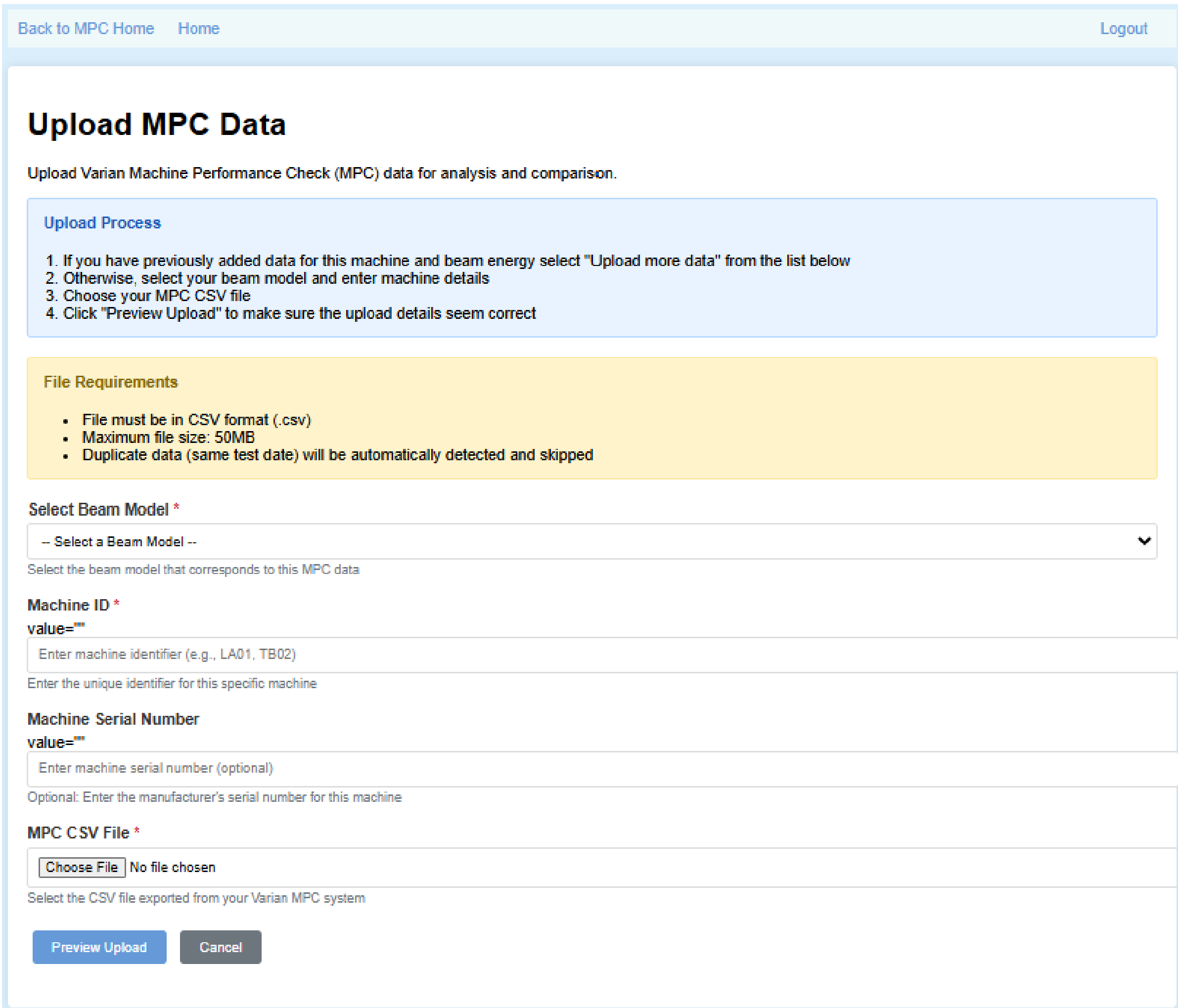

Select your beam model from the drop down menu, if you have not yet created the required beam model you will need to do this by navigating back to the home page and clicking create new beam model. Note, a beam model can be assigned to mutliple machines, the machine serial number is what is important. The machine ID is mostly there so it's easier for users to identify the machine.

Once you hit upload you will be asked to preview the upload. Please make sure the details seem correct.

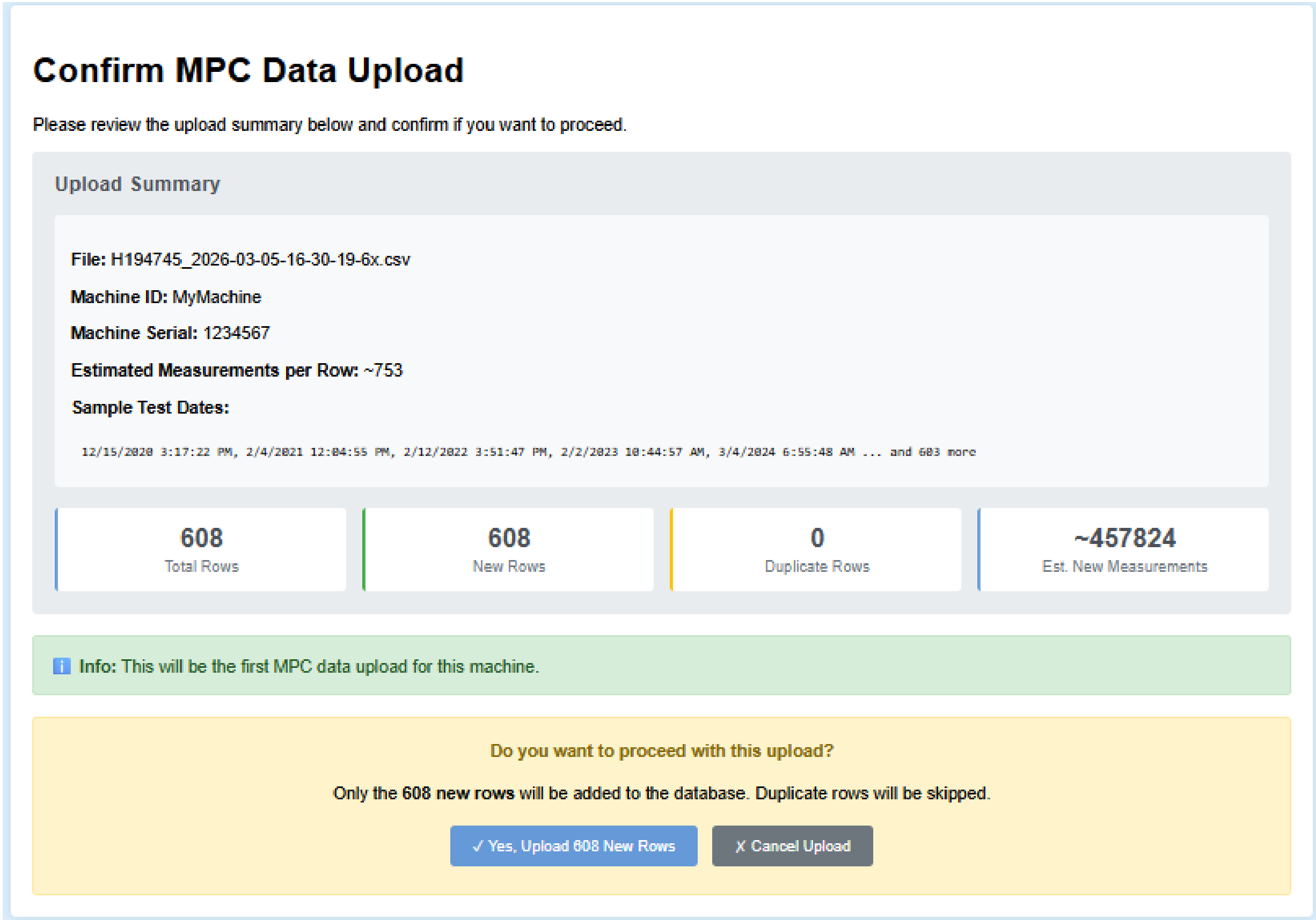

Once you hit upload **it might take a minute** to process the data.

You will see a read out of the number of new rows of data being added and the duplicate rows. As this was the first submission, all data was new.

If you add more data to an existing machine it will tell you how many new rows are being added:

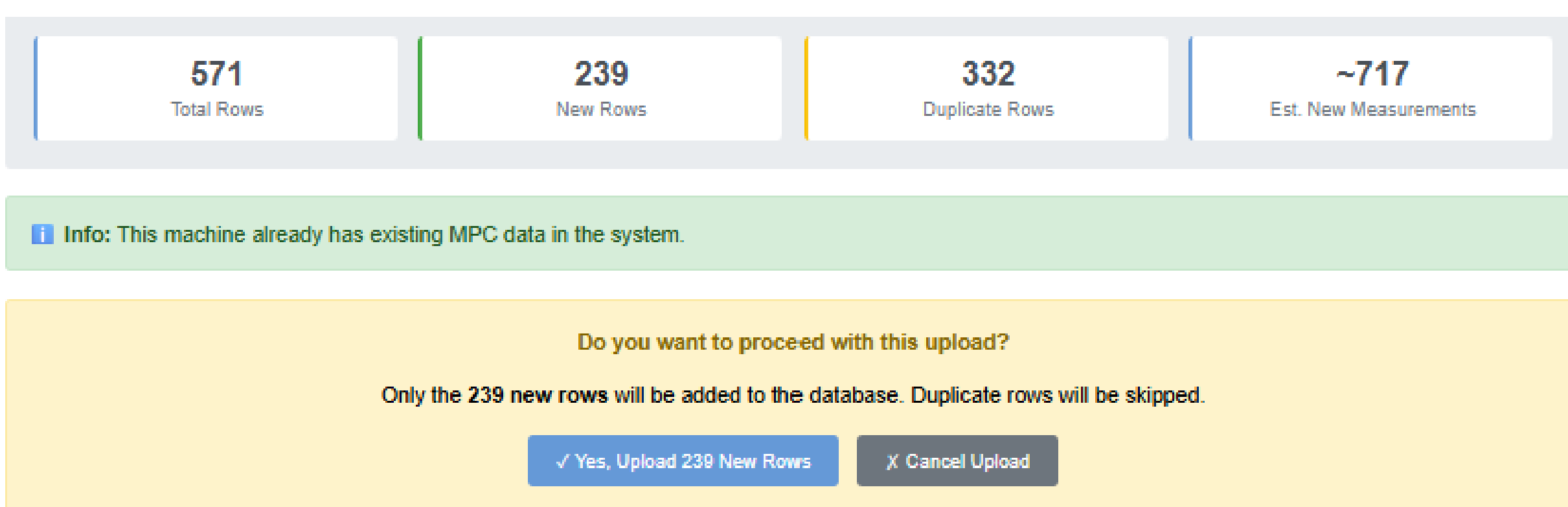

If you are adding data to an existing machine but there are no duplicate rows there will be a warning stating that existing data was expected:

⚠ **Warning:** Warning: No duplicate data found but this machine has existing data. Please verify you're uploading to the correct machine.

If the beam model energy doesn't match the filename you will get a warning, but you can override this and submit anyway (e.g. if the filename was edited but it is the correct file):

⚠ **Warning:** Warning: Expected energy '6xFFF' not found in filename for selected beam model (6FFF). Please verify the correct beam model is selected or that the file correspond to the selected energy.

If you select Upload more data to this machine the machine ID, beam model and serial number will prefill:

Your MPC Data

Previously uploaded MPC data for machines at your centre:

Machine ID	Machine Serial	Beam Model	Energy	Upload Date	Actions
MyMachine	1234567	Eclipse v18	6FFF	2026-04-01	Upload more data to this machine -->
MyMachine	1234567	Eclipse <v18	6MV	2026-04-01	Upload more data to this machine -->

Comparing data

Navigate back to the MPC Home and click **Compare MPC Data.** Select a machine and energy to see the available data you can compare. Note there are more options for the 6x beam compared to the others:

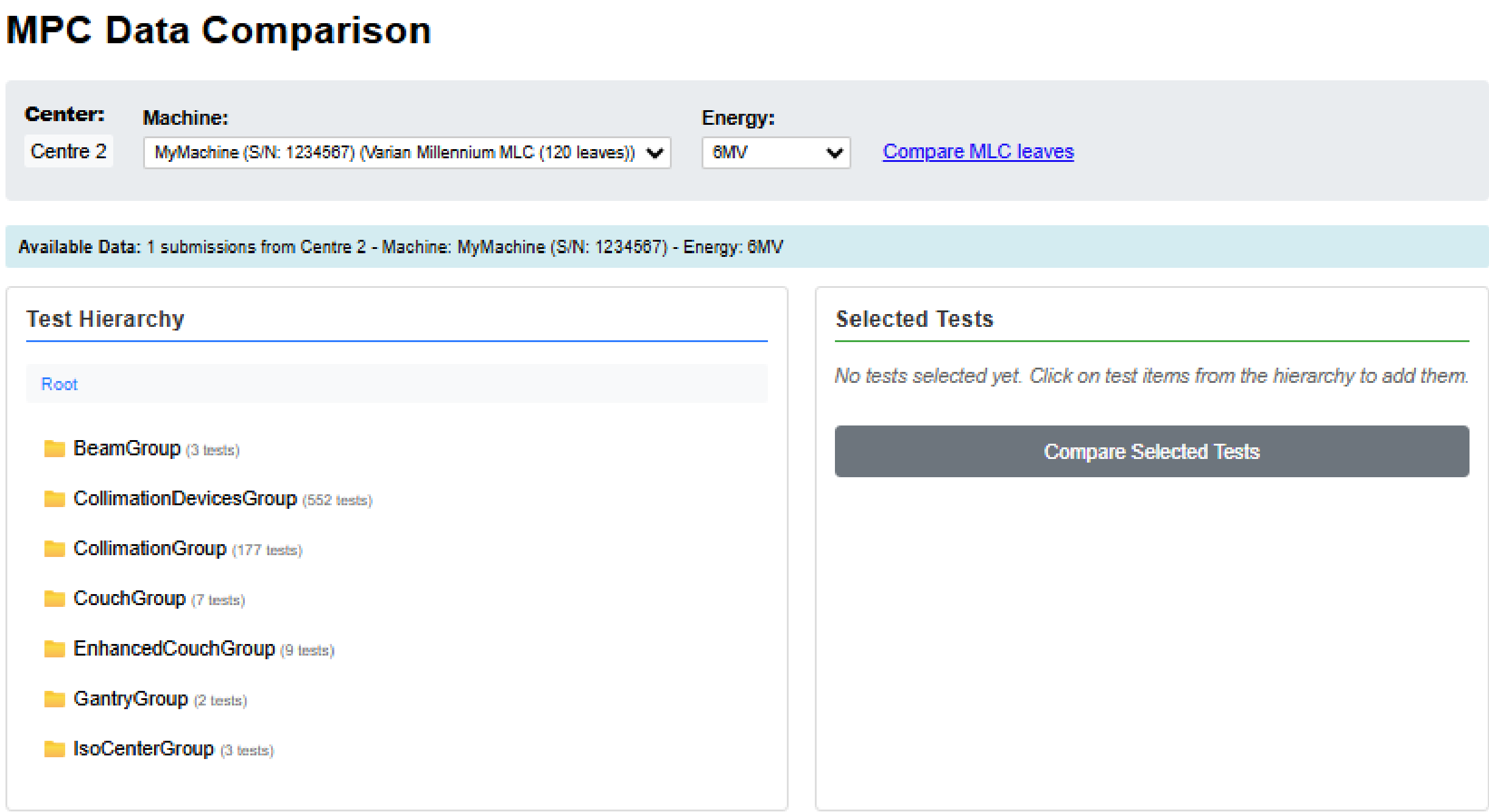

Navigate down to the test you want to compare and select it, then hit **Compare Selected Tests**.

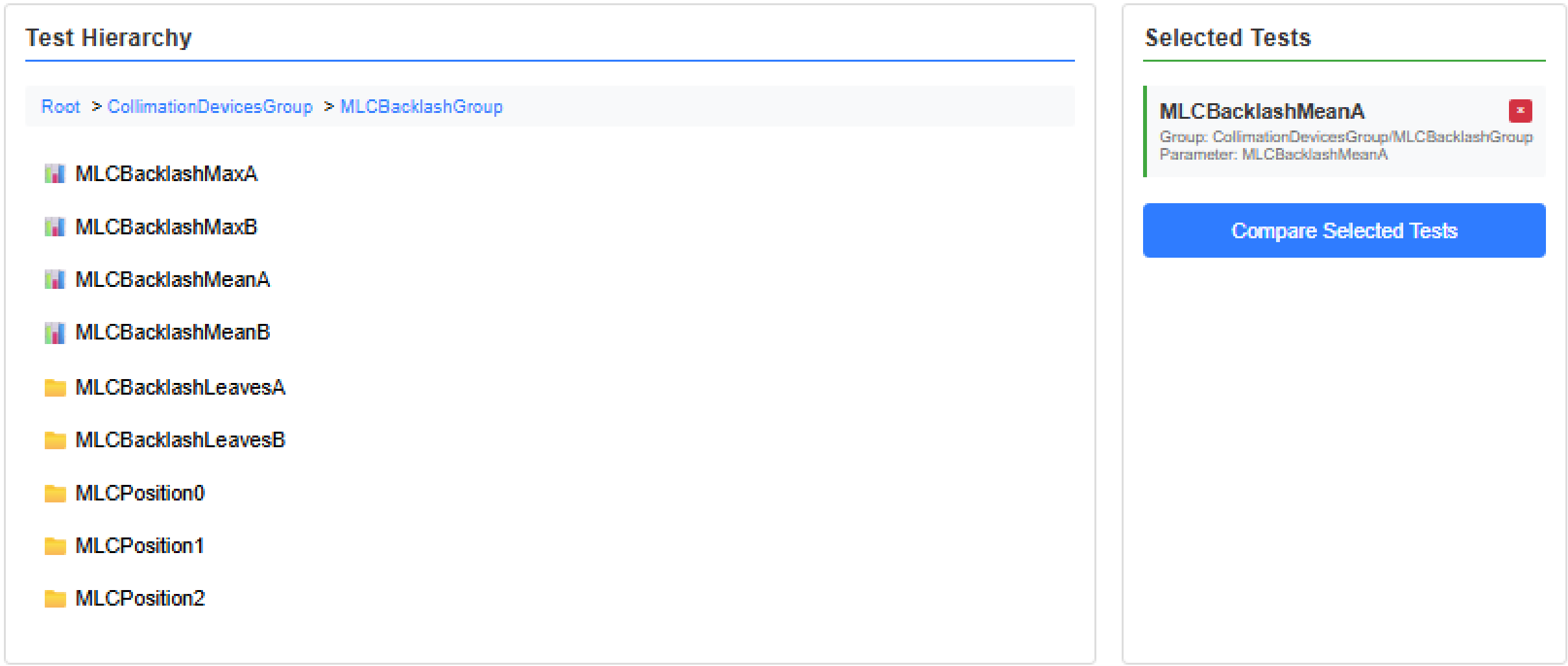

You will see soemthing like the below image. This is beam centre shift over time, the blue dots are individual measurements. The horizontal lines represent SPC analysis data. The blue lines are data for this machine and the yellow are averages for the entire cohort with matching linac and MLC model and energy. Blue solid line is the central line (mean value) for this machine, the dashed lines are the upper and lower control limits for this machine. The yellow solid line is the average central line for the cohort and the yellow dashed lines are the average upper and lower control limits for the cohort.

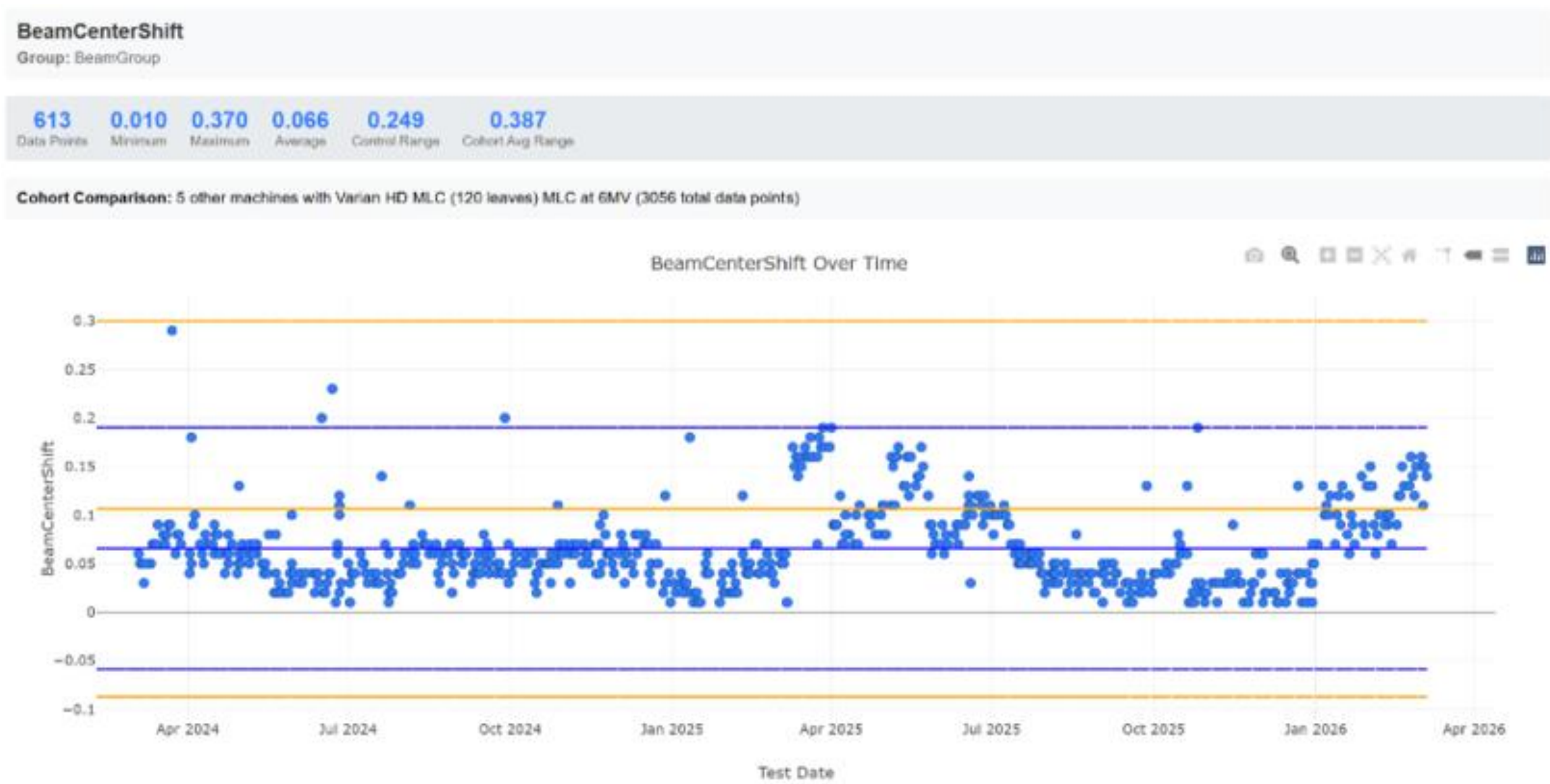

Also notice at the top there are some stats including the control range(UCL-LCL) for this machine next to the cohort average range. In this case this machine has a smaller range than the average from the cohort.

If you navigate back to select another test you have to pick the machine and energy again. But there is also another button here for **Compare MLC leaves.**

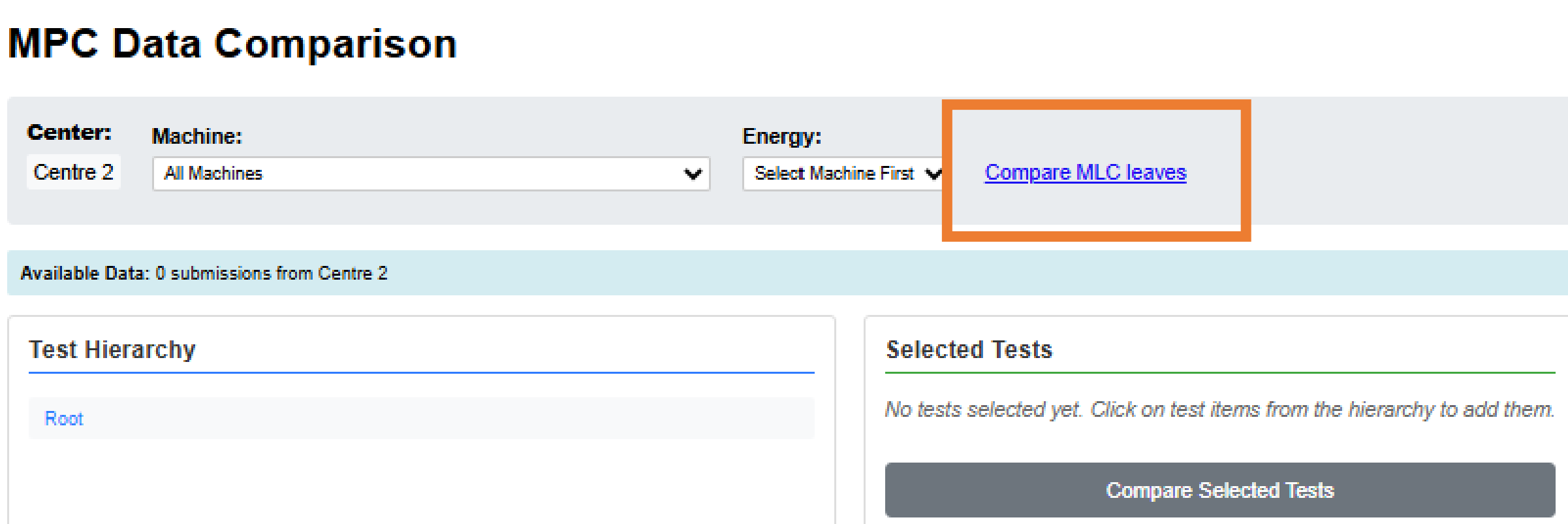

This button takes you to a page to compare indiivdual MLC leaves. Select your machine and energy (only 6x is relevant for this) and it will show the available tests to compare.

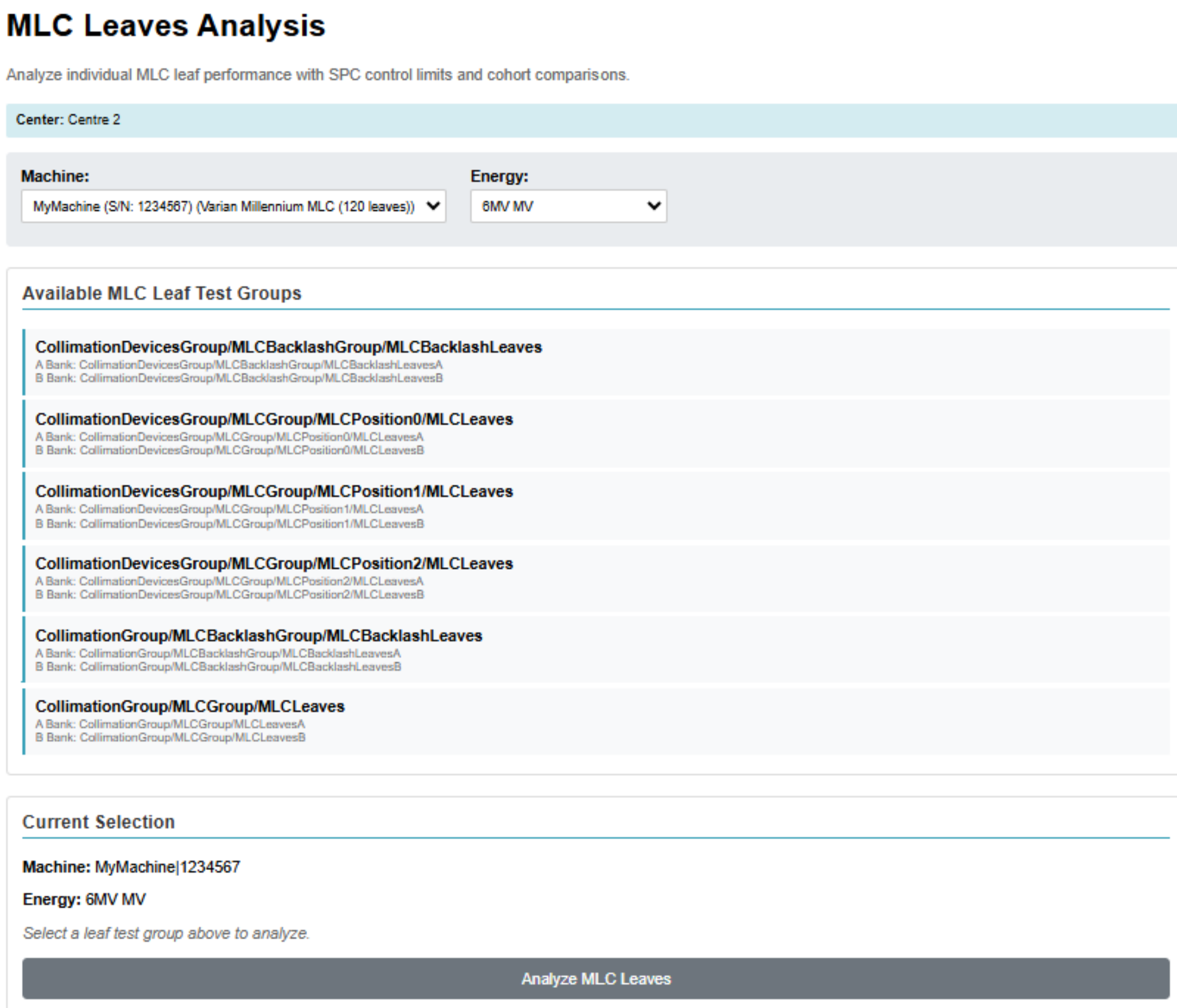

You will see something like this.

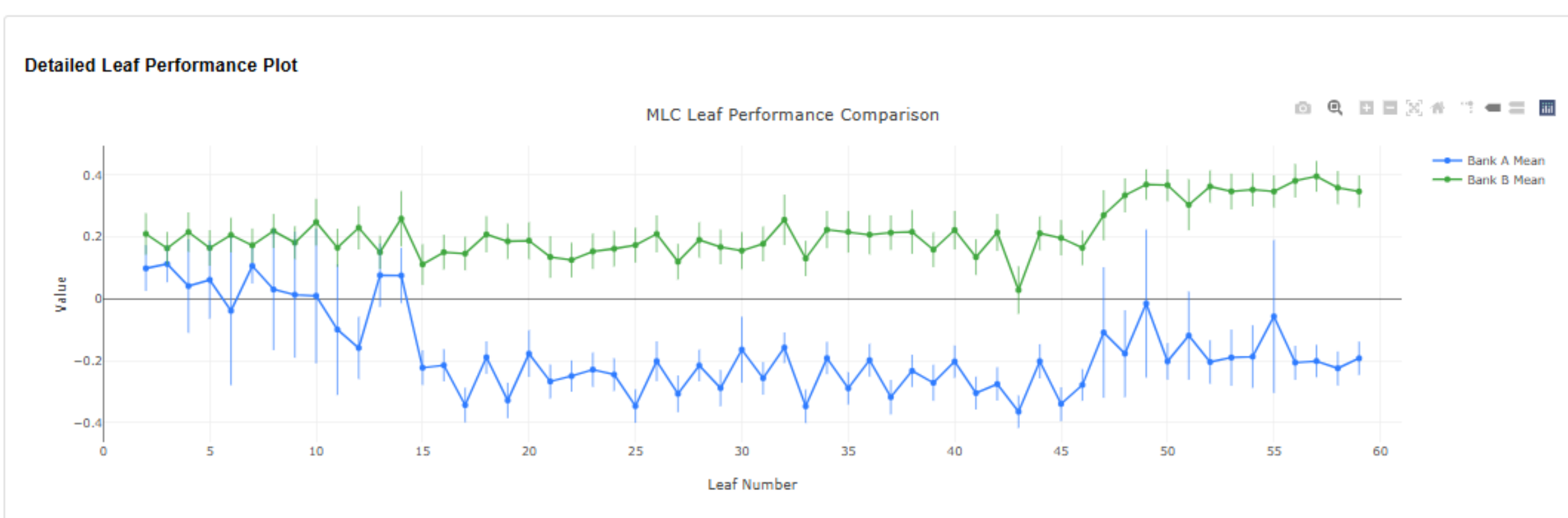

The bars on each MLC leaf are the UCL and LCL for that individual leaf. The idea being that you can look and see that, for example, leaf number 55 for bank A has a larger control range than most other leaves and go look at the data for that individual leaf.
