# Supplementary material for: Linac performance benchmarking: Feasibility of a web‐based tool for comparison of local machine performance check (MPC) results with the global community
Source: J Appl Clin Med Phys. 2026 Oct 28;27(11):e70892. doi: 10.1002/acm2.70892 (PMC13715505; doi:10.1002/acm2.70892)
Supplement: Supplementary file 2 [file ACM2-27-e70892-s001.docx]

**Supplementary Materials**

Table S-1. Summary of machines included in the study.

| Machine | MLC Model | Install Year | Center ID | Country of origin | Data collection period |
| --- | --- | --- | --- | --- | --- |
| TrueBeam | Millennium | 2016 | Center 1 | UK | Since installation |
| TrueBeam | Millennium | 2016 | Center 1 | UK | Since installation |
| TrueBeam | Millennium | 2016 | Center 1 | UK | Since installation |
| TrueBeam | Millennium | 2016 | Center 1 | UK | Since installation |
| TrueBeam | Millennium | 2016 | Center 1 | UK | Since installation |
| TrueBeam | Millennium | 2016 | Center 1 | UK | Since installation |
| TrueBeam | Millennium | 2020 | Center 2 | Australia | Two most recent years |
| TrueBeam | Millennium | 2021 | Center 2 | Australia | Two most recent years |
| TrueBeam | Millennium | 2021 | Center 3 | Australia | Two most recent years |
| TrueBeam | Millennium | 2022 | Center 4 | Australia | Two most recent years |
| TrueBeam | Millennium | 2022 | Center 5 | Australia | Two most recent years |
| TrueBeam | Millennium | 2023 | Center 6 | Australia | Two most recent years |
| TrueBeam | Millennium | 2024 | Center 1 | UK | Since installation |
| TrueBeam | Millennium | 2024 | Center 6 | Australia | Two most recent years |
| TrueBeam | Millennium | 2025 | Center 1 | UK | Since installation |
| TrueBeam | HD120 | 2016 | Center 1 | UK | Since installation |
| TrueBeam | HD120 | 2020 | Center 6 | Australia | Two most recent years |
| TrueBeam | HD120 | 2020 | Center 2 | Australia | Two most recent years |
| TrueBeam | HD120 | 2022 | Center 7 | Australia | Two most recent years |
| TrueBeam | HD120 | 2024 | Center 2 | Australia | Two most recent years |
| TrueBeam | HD120 | 2024 | Center 2 | Australia | Two most recent years |
| Halcyon | Dual layer | 2024 | Center 5 | Australia | Two most recent years |

Table S-2. Collated suggestions for tool improvements based on open-ended feedback responses.

| **Centre ID(s)** | **Suggestion** |
| --- | --- |
| Center 1 | Add tolerance limits to plots |
| Centers 1, 2 and 4 | Allow users to edit and delete submissions |
| Centers 1 and 2 | Add electrons |
| Center 2 | Simplify selection of beam models |
| Center 1 and 2 | Prescribe the serial number format entered |
| Center 1 | Automatically highlight the parameters which are outside normal data |
| Center 1 | Compare multiple machines at once |
| Center 1 | Compare amongst machines for a user's own center only |
| Center 1 | Exclude some data from analysis e.g. data from testing or a know mistake |
| Center 2 | Handle maintenance events |
| Center 1 | Upload all energies at once |
